# State Reporting Requirements for Involuntary Holds, Court-Ordered Guardianship, and the US National Firearm Background Check System

**DOI:** 10.1001/jamahealthforum.2023.3945

**Published:** 2023-11-17

**Authors:** Marian E. Betz, Deirdre M. Bowen, Ali Rowhani-Rahbar, Alexander D. McCourt, Frederick P. Rivara

**Affiliations:** 1 Department of Emergency Medicine, School of Medicine, University of Colorado Anschutz Medical Campus, Aurora; 2 VA Eastern Colorado Geriatric Research Education and Clinical Center, Denver; 3 Seattle University School of Law, Seattle, Washington; 4 Firearm Injury and Policy Research Program, University of Washington, Seattle; 5 Department of Pediatrics, School of Medicine, University of Washington, Seattle; 6 Department of Epidemiology, School of Public Health, University of Washington, Seattle; 7 Department of Health Policy and Management, Johns Hopkins Bloomberg School of Public Health, Baltimore, Maryland

## Abstract

**Importance:**

Firearm violence is a leading cause of death in the US. There is broad public agreement that individuals at high risk of perpetrating violence should not be allowed to purchase or possess firearms. The National Instant Criminal Background Check System (NICS) is a national system intended to stop firearm purchases or possession by prohibited persons, but there is heterogeneity in how states interact with and report data to NICS.

**Objective:**

To examine variability in state requirements for NICS reporting for mental health prohibitions.

**Design and Setting:**

This cross-sectional study in the US reviewed current statutes for all 50 states and the District of Columbia (enacted through the 2021 legislative session) regarding reporting mental health prohibitions to NICS or state systems. Data were analyzed from October 2022 to April 2023.

**Main Outcomes and Measures:**

For each state, the following were recorded: reliance on Federal Bureau of Investigation vs state point of contact for NICS checks; regulation requiring or allowing reporting to NICS for 3 hypothetical cases; and details on reporting responsibility and time frame. The number of NICS listings for adjudicated mental health reasons was obtained, and state rates per 1000 population were calculated. To compare states, we developed 3 hypothetical cases of individuals with risk of firearm violence, whether possibly temporary (involuntary commitment for decompensated psychosis, or involuntary short-term psychiatric hold for suicidal ideation) or chronic and progressive (court-appointed guardianship for dementia).

**Results:**

A total of 39 states required and 5 allowed reporting to NICS for court-ordered, involuntary psychiatric hospitalization. Thirteen states required and 5 allowed reporting to NICS when a court determines an individual is mentally incompetent to manage their affairs (with or without guardianship). Two states required NICS reporting for short-term emergency psychiatric holds. Five states and the District of Columbia had no legislation explicitly requiring or allowing NICS reporting in the 3 scenarios.

**Conclusions and Relevance:**

In this cross-sectional study of state laws, there was substantial heterogeneity in NICS reporting requirements and lack of clarity around processes. This raises questions about the ability of NICS to be used to block firearm purchases or possession by individuals with court-identified high risk of perpetrating violence toward themselves or others.

## Key Points

**Question:**

How do states report mental health prohibitions for firearm possession to the National Instant Criminal Background Check System (NICS)?

**Findings:**

This cross-sectional study examined state statutes for NICS reporting. When an individual has a court-ordered involuntary psychiatric commitment, 39 states required and 5 allowed NICS reporting; when an individual is court-identified as mentally incompetent to manage their affairs (with or without guardianship), 13 states required and 5 allowed NICS reporting; and when an individual is placed on a short-term emergency psychiatric hold, 2 states required NICS reporting.

**Meaning:**

Heterogeneity in reporting requirements means that the NICS may not prevent firearm possession by individuals with court-identified high risk of perpetrating violence toward themselves or others.

## Teaser

This cross-sectional study examines variability in state requirements for National Instant Criminal Background Check System reporting for mental health–related prohibitions on firearm possession in the US.

## Introduction

Firearm-related injury and death are major public health problems in the US. In 2021, 48 830 firearm-related deaths occurred.^1^ In 2022, firearms were the leading cause of death among US youth aged 0 to 24 years.^2^ Different types of firearm injuries and deaths exist (including interpersonal violence, suicide, unintentional shootings, legal intervention, and mass shootings), with differing risk factors and rates across demographic groups and geographic regions.^3^

Across types of firearm violence and populations, a common approach is limiting firearm purchase or possession by certain individuals at certain times, such as when they pose a significant, imminent risk to themselves or others. The 1993 federal background check statute (the Brady Handgun Violence Prevention Act), which pertains to checks performed at the time of firearm acquisition, aims to prevent firearm possession by certain individuals, including those with mental health conditions. A prohibition exists on firearm sale or transfer of a firearm to a person who “has been adjudicated as a mental defective or has been committed to any mental institution.”^4^ The antiquated term “mental defective” is defined by the American Psychological Association as a “descriptor for persons who have a mental disorder or neurological deficit that renders them incapable of appraising the nature of their conduct”^5^; states interpret the term in practice. Increasingly, Extreme Risk Protection Order (ERPO) laws are another mechanism to block individuals with imminent risk of harm to self or others from accessing firearms; as of summer 2023, 21 states and the District of Columbia (DC) have ERPO-type laws.^6^ Short-term emergency commitment laws, sometimes called 72-hour holds or psychiatric holds, are present in all states; these laws allow involuntary detention and treatment of individuals with imminent risk and thereby may prevent firearm access.^7,8,9^

The federal Brady Act also established the National Instant Criminal Background Check System (NICS). It began in 1998 as a national system intended to help stop the sale or transfer of firearms to individuals prohibited from purchasing or possessing guns. In most states, federally licensed dealers contact the Federal Bureau of Investigation (FBI) for a background check through NICS, but some states require certain dealers to contact state or local agencies.^10^ NICS includes statutorily defined firearm possession prohibition categories (eg, felony conviction, domestic violence restraining order, convicted of domestic violence misdemeanor), including adjudicated mental health. This issue is relevant to physicians, other clinicians caring for such patients, their families, and the general community.

Recently, we completed a high-level review of state laws related to when, to whom, and by whom mental health information must be reported to NICS.^11^ Here, we sought to highlight the clinical relevance of this information through 3 case examples to examine state requirement variability. We chose cases where an individual would not be safe to possess or purchase a firearm due to mental or cognitive incapacity and risk of harm to self or others; 2 cases are potentially temporary conditions and 1 is chronic and progressive.

## Methods

As described elsewhere,^11^ we used the Thomson Reuters Westlaw database to catalog state mental health reporting laws on firearm prohibitions and background checks in each of the 50 states. Laws enacted through the 2021 legislative sessions were included. Five research assistants with legal training conducted the search and created the data set; a faculty author (D.M.B.) reviewed findings from all 50 states and DC. The search documented each state’s statutory and regulatory language concerning mental health reporting; additional abstracted data included the type of mental health events that must be reported, as well as to whom, on what timeline, and by whom they are reported. For each state and DC, we obtained public data for the number of persons listed in NICS Indices for adjudicated mental health as of January 3, 2023,^12^ and we calculated state rates using US Census Bureau population estimates.^13^ This study of state legislation and state-level, publicly available data was not human participant research and did not require review by the Colorado Multiple Institutional Review Board. We followed Strengthening the Reporting of Observational Studies in Epidemiology (STROBE) reporting guidelines when applicable.^14^

We also developed 3 hypothetical cases where an individual cannot safely have firearm access, temporarily (case 1 and 3) or permanently (case 2). For each case, we examined reporting requirements in all states and chose 4 states to highlight variation in state-level approaches.

### Case 1. Court-Adjudicated Involuntary Psychiatric Hospitalization for Decompensated Psychosis

A family brings a 35-year-old man to an emergency department (ED) for paranoia and auditory hallucinations telling him to kill government officials, who he believes are monitoring him. The patient has a history of posttraumatic stress disorder and psychosis. He says he stopped taking his prescribed medications and plans to buy firearms. After medical evaluation, he is involuntarily admitted to a psychiatric facility for stabilization, including restarting his prescribed medications. At the psychiatric facility, he remains in a psychotic state and is unwilling to stay; his treating clinicians, with his family’s support, apply to a court for and are granted involuntary commitment for ongoing stabilization.

### Case 2. Court-Adjudication of Incompetence (With Guardianship) Due to Dementia

A 75-year-old woman has Alzheimer dementia and progressive memory problems. She lives alone and refuses to move in with her son or to a facility. Her son is increasingly concerned that she cannot care for herself; she rarely bathes or obtains fresh food, she takes her medications erratically, she threatens neighbors with her shotgun, and recently she nearly hit a pedestrian with her car. He petitions for and, after a court hearing, is granted guardianship over her.

### Case 3. Involuntary Short-Term Emergency Hold for Suicidal Ideation

A 55-year-old man is brought by ambulance to an ED for suicidal ideation. His wife called 911 after he threatened to shoot himself with his handgun. He was reluctant to go to the hospital, so the police placed him on an involuntary short-term hold. In the ED, the medical team and a behavioral health specialist evaluate him. He reveals a history of depression being treated by his primary care physician and an outpatient therapist. He fought with his wife about their finances, which made him wonder if she would be better off without him. After an ED evaluation, his suicide risk has lowered: he says he wants to live, has identified ways to reduce financial stressors, has made up with his wife, and has a next-day appointment with his therapist. His wife has moved their handgun to a secure out-of-home storage location. The involuntary hold is lifted, and he is discharged home with his wife.

## Results

### Reporting Requirements

**Figure 1.**
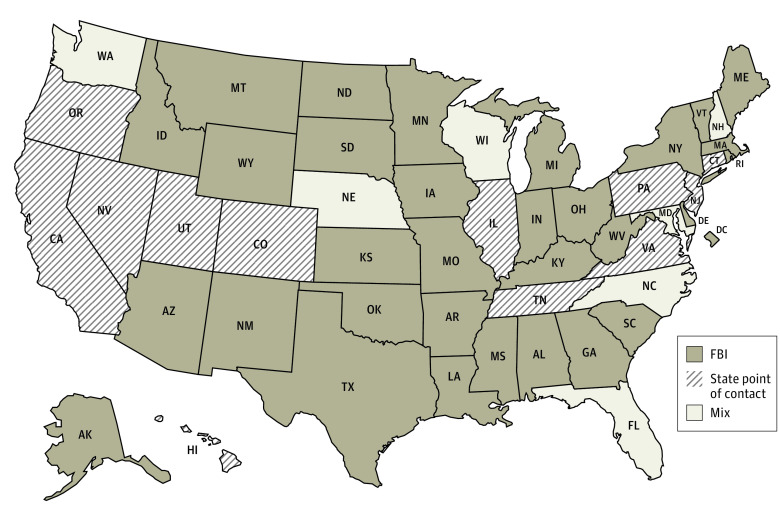
States Using Federal Bureau of Investigation (FBI), State Point of Contact, or a Mix for National Instant Criminal Background Check System Checks Florida uses a state system for all firearms except certain pawn transactions; Maryland, Nebraska, New Hampshire, North Carolina, Washington, and Wisconsin use a state point of contact for handguns and FBI for long guns.^10^

**Figure 2.**
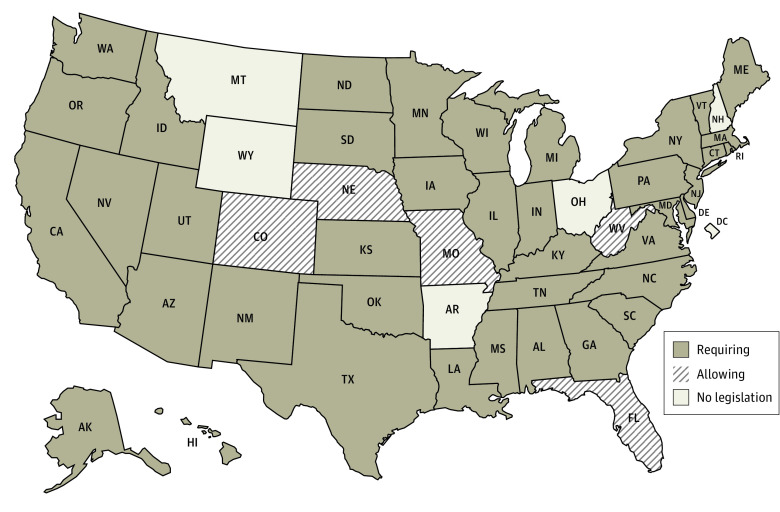
States Requiring or Allowing Reporting to National Instant Criminal Background Check System for Involuntary Inpatient Commitment After Court Hearing Two states required reporting only when an individual had been committed for 14 days (Washington) or 30 days (Maryland) or more of hospitalization.^10^

**Figure 3.**
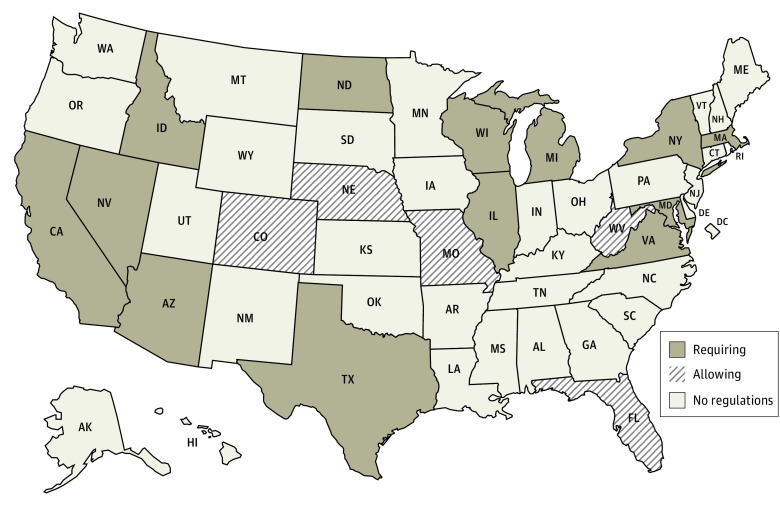
States Requiring or Allowing Reporting to National Instant Criminal Background Check System for Court Adjudication That an Individual Is No Longer Competent to Manage Their Affairs (May Include Guardian Appointment)

A total of 32 states and DC used the FBI for all NICS checks, 12 states used a state system, and 6 states used a mix of the FBI and a state system (Figure 1).^10^ States varied in their NICS reporting requirements and logistics (Table, Figures 2 and 3).^10^

**Table.** Reporting Responsibility and Time Frame for States That Require (R) or Allow (A) National Instant Criminal Background Check System (NICS) Reporting After Court-Ordered Involuntary Commitment (IC), Adjudication as Incompetent (May Include Guardian Appointment; I/G), or 72-Hour Involuntary Hold (IH)

State	IC^a^	I/G^b^	IH^c^	Who reports to NICS	Time frame	Citation(s)^d^
Alabama	R			Court to state law enforcement agency	Immediately/as soon as possible	Ala Code 1975 §22-52-10.8
Alaska	R			Court to Department of Public Safety	Immediately	Alaska Stat §44.41.045(a)
Arizona	R	R		Court to Supreme Court to Department of Public Safety	Not specified	AZ ST §36-540
Arkansas				NA	NA	NA
California	R	R	R	Court to State Department of Justice	Within 1 court day	CA WEL & INST §8103
Colorado	A	A		State Court Administrator	48 h	Colo Rev Stat §§13-5-142(1)-(3); 13-9-123(1)-(3)
Connecticut	R			Department of Emergency Services and Public Protection, Department of Mental Health and Addiction Services and Judicial Department	Without delay	Conn Gen Stat §29-36l(d)(2)
Delaware	R			Delaware Psychiatric Center and any other hospital defined under §5001(9) (and responsible persons) to the State Bureau of Identification	Not specified	DE ST TI 16 §5161(14) and TI 11 §5009
District of Columbia				NA	NA	NA
Florida	A	A		Court to Department of Law Enforcement	Within 30 d after court ruling	Fla Stat §790.065(2)(a)(4)
Georgia	R			Superior and/or probate courts to Georgia Crime Information Center	At a time agreed on with Georgia Bureau of Investigation	Ga Code Ann §35-3-34(e)(2)
Hawaii	R			Court to state Criminal Justice Data Center	Not specified	HI ST §560:5-311(d)
Idaho	R	R		Court to state police	Not specified	§560:5-311. Findings; order of appointment
Illinois	R	R		Circuit Court Clerk to state police; school administrator to state police; physician, clinical psychologist, or qualified examiner to Department of Human Services to state police	Within 7 d of court order; all others 24 h	405 Ill. Comp Stat 5/6-103.1; 405 ILCS 5/6-103.3
Indiana	R			Court to Office of Judicial Administration	Not specified	Ind Code §33-24-6-3(a)(8)
Iowa	R			Court to Department of Public Safety	Not specified	Iowa Code §724.31(1)
Kansas	R			Court to state Bureau of Investigation	Within 5 d after receipt of order; immediate NICS entry by Bureau of Investigation	Kan Stat Ann §59-2966 (West); Kan Stat Ann §75-7c25 (West)
Kentucky	R			Court to state police	Not specified	Ky Rev Stat Ann §237.108(1)
Louisiana	R			Court to Supreme Court	Within 10 business days to Supreme Court, then within 15 business days to NICS	La Stat Ann §13:753
Maine	R			Court to Department of Public Safety	Not specified	Me Stat 25 §1541(3)(C)
Maryland	R	R		Facility where person is committed; Maryland court	Within 10 d from facility; promptly from court	Md Code Ann, Pub Safety §5-133.2(b)
Massachusetts	R	R		Court	On ordering the commitment	Mass Gen Laws ch 123 §§35; 36C
Michigan	R	R		Court to state police	Immediately	Mich Comp Laws Serv §330.1464a(1)
Minnesota	R			Court to Commissioner of Human Services	Immediately to commissioner; by request to law enforcement agencies	Minn Stat §253B.09, subd 9
Mississippi	R			Court	Within 30 d after court ruling	MISS CODE ANN §45-9-103(1)
Missouri	A	A		Clerk of the Court to the Department of Mental Health to State Highway Patrol	Without undue delay	Mo Rev Stat §552.030.7
Montana				NA	NA	NA
Nebraska	A	A		Court to Department of Health and Human Services and Nebraska state patrol	As soon as possible, within 30 d after order	Neb Rev Stat §69-2409.01
New Hampshire				NA	NA	NA
Nevada	R	R		Court and Central Repository	Within 5 business days	Nev Rev Stat §§174.035; 175.533; 175.539; 178.425; 179A.163
New Jersey	R			Court Office of Administration and State Superintendent of Police	Not specified	NJ ST 30:4-24.3a
New Mexico	R			Court administrative office	As soon as possible, within 10 d of receipt of information	NM Stat Ann §34-9-19
New York	R	R		Four groups of mental health professionals to county mental health officials to Commissioner of Mental Health to New York State Division of Criminal Justice Service	As soon as practicable from health practitioner	NY Mental Hyg Law §9.46 (McKinney) section (b); NY Mental Hyg Law §7.09 (McKinney) §§(j)(1)-(2)
North Carolina	R			Court	Within 48 h (business days only) of receiving notice of court order	NC Gen Stat Ann §14-409.43
North Dakota	R	R		Court	Not specified	ND Cent Code Ann §62.1-02-01.2
Ohio				NA	NA	NA
Oklahoma	R			Court	As soon as possible, within 10 business days after court order	Okla Stat Ann 21 §1290.27
Oregon	R			Court to county sheriff	Not specified	Or Rev Stat Ann §426.133; Or Rev Stat Ann §426.130
Pennsylvania	R			Court or “mental health review officer or county mental health and mental retardation administrator”^15^ to sheriff	Within 7 d of the commitment or adjudication	18 Pa Stat and Cons Stat Ann §6109; 50 Pa Stat Ann §7111 subsection (b)
Rhode Island	R			Court	Within 48 h or court order	40.1 RI Gen Laws Ann §40.1-5-8
South Carolina	R			Court to state Law Enforcement Division	Within 5 d from the filing of each order	SC Code Ann §23-31-1020
South Dakota	R			Prosecuting attorney or board of mental illness to state Attorney General	Within 7 working days after verdict or adjudication or determination	SD Codified Laws §23-7-47
Tennessee	R			Court	As soon as practicable, but no later than the third business day	Tenn Code Ann §16-1-117
Texas	R	R		Court to Department of Public Safety	Within 30 d of the court date	TN ST 16-10-213
Utah	R			Court to state Bureau of Criminal Identification	Within 48 h of the determination, and then within 48 of receiving the record	UT ST 53-10-208.1(2); UT ST 53-10-213
Vermont	R			Court	Within 48 h of court order	VT ST T 13 4824(a)
Virginia	R	R		Court to Central Criminal Records Exchange	As soon as practicable but not later than the close of business the following business day	VA ST 37.2-819(B)
Washington	R		R	Court to Department of Licensing	Within 3 judicial days after order	RCW 9.41.047(1)(b); Wash Rev Code Ann §71.05.182
West Virginia	A	A		Court to central state mental health registry	As soon as practicable to state registry, then not specified to NICS	WVa Code §61-7A-3(b)
Wisconsin	R	R		Court to state Department of Justice	In a timely manner	WI ST 51.20(13); 51.45; 54.10; 55.12
Wyoming				NA	NA	NA

Abbreviation: NA, not applicable.

^a^ IC refers to NICS reporting after involuntary inpatient commitment after court hearing.

^b^ I/G refers to NICS reporting after court adjudication that an individual is no longer competent to manage their affairs (may include guardian appointment).

^c^ IH refers to NICS reporting after 72-hour involuntary hold.

^d^ See state statutes for full details.

For the first hypothetical case (court-ordered involuntary psychiatric commitment), 5 states (Arkansas, Montana, New Hampshire, Ohio, Wyoming) and DC had no legislation explicitly requiring or allowing NICS reporting (Table, Figure 2). Five states (Colorado, Florida, Missouri, Nebraska, West Virginia) explicitly allowed, but did not require, NICS reporting. The remaining 40 states required NICS reporting, albeit with different mechanisms and timelines (Table).

For the second hypothetical case (court determination of incompetence to manage affairs, with or without guardianship), fewer states had regulations concerning NICS reporting (Table, Figure 3). Five states allowed but did not require reporting (Colorado, Florida, Missouri, Nebraska, West Virginia), and 13 states required reporting (Arizona, California, Idaho, Illinois, Maryland, Massachusetts, Michigan, Nevada, New York, North Dakota, Texas, Virginia, Wisconsin). The remaining 32 states and DC had no regulations explicitly requiring or allowing reporting.

For the third hypothetical case (involuntary short-term hold for suicide risk), 2 states (California, Washington) required NICS reporting (Table). The remaining 48 states and DC had no legislation specifically allowing or prohibiting NICS reporting.

**Figure 4.**
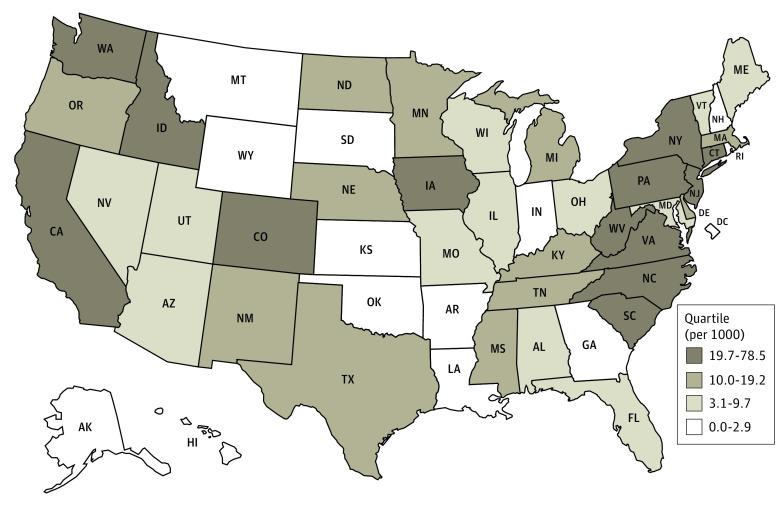
State Rate of Individuals in National Instant Criminal Background Check System (NICS) for Adjudicated Mental Health Reasons, by Quartiles per 1000 Population Number of active entries in the NICS Indices as of January 3, 2023; NICS Indices contain information provided by federal, state, local, and tribal agencies on individuals prohibited from receiving firearms under federal or state law.^12^ State population denominators based on US Census Bureau estimates as of July 1, 2022.^13^

The number of current individuals listed in NICS for adjudicated mental health reasons varied across states, from 22 in Wyoming to 1 197 677 in California. The mean number per 1000 population was 14.9, with the highest rate (78.5 per 1000 population) in Pennsylvania (Figure 4).^12,13^ In the quartile of lowest rates, 5 of 13 (Arkansas, DC, New Hampshire, Montana, Wyoming) were locations with no legislation explicitly requiring or allowing reporting. The other state without specific legislation requiring or allowing reporting (Ohio) had 79 041 cases in NICS (rate, 6.7 per 1000 population; Figure 4).

### State Examples

For both court-determined cases (involuntary commitment and incompetence with or without guardianship), but not for the short-term emergency hold case, Arizona required reporting to NICS, but the process was convoluted and without a clear timeline. According to state law: “The court shall transmit [the individual’s information] to the [state] supreme court. The supreme court shall transmit the case information and the date of the incompetency finding to the department of public safety.… The department of public safety shall transmit the information to the national instant criminal background check system.”^16,17^ Arizona law had a similar procedure for restoration of rights (and removal from NICS) when an individual is “subsequently found competent,” but required reporting within 10 days.

In Ohio, for both court-adjudicated cases but not for short-term holds, reporting to the state-based system was required (by the judge or chief clinical officer) within 7 days of the court order. Ohio’s requirement reads: if “an individual is found by a court to be a mentally ill person subject to court order or becomes an involuntary patient other than one who is a patient only for purposes of observation, the probate judge who made the adjudication or a chief clinical officer of the hospital, community mental health services provider, or facility in which the person is an involuntary patient shall notify the office of the attorney general.”^18^ Ohio required the Attorney General’s office to compile these data and use them in the state system for criminal records checks and incompetency checks for applications for concealed handgun licenses.^19^ However, Ohio’s state statute neither required nor prohibited NICS reporting, so information may not be visible to other states.

Rhode Island treated the 3 hypothetical cases differently from each other. For court-ordered involuntary commitment, NICS reporting was required within 48 hours. Specifically, the district court must transmit “the name, date of birth, gender, race or ethnicity, and date of civil commitment to the National Instant Criminal Background Check System (NICS) database of all persons subject to a civil court certification order pursuant to this section within forty-eight (48) hours of certification.”^20^ For the cases involving court-appointed guardianship or short-term emergency hold, Rhode Island did not require NICS reporting.

In Texas, for either of the court-ordered hypothetical cases but not for a short-term emergency hold, the court clerk was required to report the information to the Texas Department of Public Safety within 30 days.^21^ While the state required reporting of adjudication to NICS, the statute did not clarify how or within what time frame the Department of Public Safety must do this reporting.^21^

## Discussion

Firearm policy remains politically divisive in the US,^22^ yet broad bipartisan support exists for restricting gun access among certain individuals who are at risk. A 2021 survey found that 85% of Republicans and 90% of Democrats supported “preventing those with mental illnesses from purchasing guns.”^23^ We chose 3 cases to analyze in which an individual had impairment and risk of harm to self or others. Two cases (decompensated chronic psychosis and acute suicidal ideation) represented potentially temporary conditions, and the other (dementia) a chronic and progressive condition. Only 2 of the hypothetical cases involve a court hearing because in the majority of states, police officers can initiate short-term holds.^8,24^ Our reporting requirements analysis shows the confusing heterogeneity within and across states—including both case and state requirements differences—and loopholes that might allow an at-risk individual to access firearms.

The NICS and mandatory background checks do have benefits. Prior research has demonstrated that subsequent firearm-related or violent crime was reduced among prospective purchasers who were denied sale due to misdemeanor^25^ or felony^26^ convictions. Research has also shown that adolescents in states with universal background checks were less likely to carry guns.^27^ For prohibited mental health events—the focus of our analysis—prior results are mixed on whether background check policies or mental health reporting reduces suicide or homicide rates.^28^

There have been attempts to improve NICS since it became operational in 1998.^29^ The 2007 mass shooting at Virginia Tech spurred specific discussion about reporting gaps and led to the NICS Improvement Amendments Act.^29^ The NICS Act Record Improvement Program, established in 2008, provided funding to states to improve NICS reporting; by the end of 2017, 29 states had received federal funding through the program. The number of states with laws requiring or allowing NICS reporting increased from 8 in 2007 to 43 in 2017, and there was a 9-fold increase in the number of state-submitted mental health records, to 4.9 million in 2017.^30^ The 2018 Fix NICS Act had bipartisan backing and support from firearms organizations.^31^ The act required federal agencies and states to create data reporting and implementation plans; it provided state funding for implementation efforts. Within 2 years after the bill signing, records in the NICS Indices (the primary repository of mental health reporting) increased by 19%,^32^ and states with laws that required reporting had higher reporting rates,^30^ as also shown in our study.

While continued efforts to improve the NICS offer promise, our analysis highlights questions about the best practical mechanisms for limiting firearm access in at-risk individuals. Reporting individuals to NICS should prohibit purchase or possession of firearms, yet many states do not proactively confiscate existing weapons from newly prohibited persons. Thus, clinicians and families and friends of at-risk individuals also play a role in reducing firearm access. Ideally, voluntary approaches would be tried first, with the engagement of the at-risk person; options include changing firearm storage at home (eg, locked and inaccessible to the person at risk) or temporary storage away from home.^33,34^

In some states, an involuntary option is an ERPO. In the case of decompensated psychosis or suicidal ideation, the risk may decrease once the condition is treated, so short-term restriction (eg, an ERPO) may be appropriate when voluntary efforts have failed. In any case of psychiatric illness, careful thought is needed about whether an involuntary hold or involuntary commitment is required, or whether treatment goals can be accomplished through voluntary approaches.^35^ Nearly half of the people in the US will meet diagnostic criteria for a mental illness in their lifetime (including substance use and mood disorders),^36^ but very few individuals with mental illness commit violence; when they do, it is most often self-directed.^37^ Suicidal crises often occur after only a short period of deliberation, and only 10% of those who survive suicide attempts later die by suicide^38^; it is therefore critical to recognize that suicidal crises are often brief and are survivable.^39^ While the US Supreme Court has upheld firearm prohibitions for individuals with involuntary civil commitment, long-term prohibition on firearm access for individuals with a prior mental illness history—or with a short-term emergency psychiatric hold—may be overly restrictive and stigmatizing.^40^ Currently, a Circuit Court split exists concerning lifetime vs time-limited firearm bans for involuntary commitment.^41^

In cases of long-term, progressive mental illness or cognitive impairment, including from dementia, an ERPO with renewals could still be an option, but family members could begin with voluntary approaches, including advance planning (in early stages of illness) and transfer or sale of firearms.^42,43,44^ Federal laws do not explicitly prohibit purchase or possession of firearms by individuals with dementia; as in the case example, a person with court adjudication and guardian appointment may (or may not) be reported to NICS. Only 2 states (Texas and Hawaii) have legislation restricting firearm access in dementia.^44^

For clinicians, the presented cases are relevant examples of individuals with acute firearm injury risk who may interface with the clinical system. Clinicians are generally not the individuals responsible for NICS reporting when it is required, and most states do not allow clinicians to request an ERPO. However, clinicians should be aware of the requirements and options in their state so that they can counsel patients and families. No state or federal laws prohibit clinicians from discussing firearm access with their patients; such discussions are important in cases of elevated risk.^45^ National clinician organizations support discussing firearm safety with patients and families and urge clinicians to be proactive.^46,47^ This is especially important because even reporting mental health events and conditions to NICS may not result in the removal of firearms from a home.

### Limitations

Limitations of this study include that we did not examine actual reporting practices across states or compliance with state requirements. Questions unanswered from this analysis include NICS reporting practices in cases of risk without court adjudication, such as if the individual in the first case agreed to a voluntary psychiatric hospitalization without court involvement. Additional work is needed to examine actual NICS reporting practices, including how often information on prohibited persons is updated in NICS or the use of processes for subsequent removal from NICS (eg, after resolution of risk).

## Conclusions

In this cross-sectional study of state laws, findings demonstrated substantial heterogeneity in NICS reporting requirements and lack of clarity around processes. Advocates for either more permissive or restrictive firearm laws agree that individuals with substantial mental illness or cognitive impairment should not have access to firearms, at least during at-risk times. Yet despite broad public agreement about limiting firearm purchase for certain individuals with high risk of harm to themselves or others, the heterogeneity in the use of NICS and other firearm background check systems may prevent such limitations from occurring. Given the increase in mass shootings, firearm homicides, and suicides in the US and the public’s safety concerns, an opportunity for policy action exists^28^ at the state and federal level to clarify, unify, and strengthen NICS reporting requirements. Ongoing public and professional discussion is needed about what situations should prompt formal actions (eg, NICS reporting, ERPO) vs informal action. In particular, we need to ensure that mental health–related restrictions do not become so stringent that they dissuade individuals from seeking care.

## Notes

Supplement.
Data Sharing Statement
