# Errors in Key Points, Abstract, Results, and Table

**DOI:** 10.1001/jamahealthforum.2023.4904

**Published:** 2024-01-26

The Original Investigation titled “State Reporting Requirements for Involuntary Holds, Court-Ordered Guardianship, and the US National Firearm Background Check System,”^1^ published November 17, 2023, included an error in the number of states reported as requiring National Instant Criminal Background Check System (NICS) reporting for court-ordered involuntary psychiatric commitment. The number has been corrected from 39 to 40 in the Key Points and Abstract. Also, the number of states that required NICS reporting when an individual is placed on a short-term emergency psychiatric hold was changed from 2 to 1 (omitting California) in the Key Points, Abstract, Results, and Table. This article has been corrected online.
