# Supplementary material for: State Reporting Requirements for Involuntary Holds, Court-Ordered Guardianship, and the US National Firearm Background Check System
Source: JAMA Health Forum. 2023 Nov 17;4(11):e233945. doi: 10.1001/jamahealthforum.2023.3945 (PMC10656649; doi:10.1001/jamahealthforum.2023.3945)
Supplement: Supplement. — Data Sharing Statement [file jamahealthforum-e233945-s001.pdf]

Data Sharing Statement

Betz. State Reporting Requirements for Involuntary Holds, Court-Ordered Guardianship, and the US National Firearm Background Check System. *JAMA Health Forum*. Published November 17, 2023. doi:10.1001/jamahealthforum.2023.3945

Data
Data available: Yes
Data types: Data (not involving human participants)
How to access data: dbowen@seattleu.edu
When available: With publication

Supporting Documents
Document types: None

Additional Information
Who can access the data: researchers whose proposed use of the data has been approved
Types of analyses: for any purpose
Mechanisms of data availability: after approval of a proposal
